# Analyzing the clinical characteristics of the SCAMP5 gene in gliomas and establishing a predictive model

**DOI:** 10.1097/MD.0000000000041147

**Published:** 2025-01-03

**Authors:** Bin Wu, Ling Zeng, Li Liu, Xianbi Tang, Yushi Zhong

**Affiliations:** a Department of Neurology, Hunan University of Medicine General Hospital, Huai Hua, China; b Department of Neurology, The Fourth Clinical College Affiliated to Jishou University, Huai Hua, China.

**Keywords:** glioma, predictive model, SCAMP5 gene

## Abstract

Gliomas are frequently occurring tumors in the nervous system. The secretory carrier membrane protein 5 (SCAMP5) plays a distinct role in the cytosolic function of mammalian cells and is associated with different neurological disorders. However, how SCAMP5 is expressed and its prognostic value in gliomas is unclear. The datasets were downloaded from the Chinese Glioma Genome Atlas website (http://www.cgga.org.cn/). We conducted a Cox survival analysis to establish a correlation between SCAMP5 gene expression and the general survival rate of patients with glioma. We performed Gene Ontology analysis to determine the biological functions of the SCAMP5 gene. Finally, we constructed a prediction model using primary relapse state type, age, grade, isocitrate dehydrogenase mutation status, 1p/19q co-deletion status, and the SCAMP5 gene expression value. Using this model, we can forecast the survival rates of patients for 1, 2, 3, 5, and 10 years. SCAMP5 was enriched in low-grade gliomas and isocitrate dehydrogenase mutant gliomas, 1p19q-deficient gliomas. SCAPM5 is an independent prognostic factor for the overall survival of glioma patients. Predictive models developed by SCAMP5 were able to predict well the long-term survival of patients. The evidence suggests that the SCAMP5 gene plays a significant function in glioma patients. There is a clear correlation between the expression of the SCAMP5 gene and the overall survival of glioma patients.

## 1. Introduction

Glioma represents the most prevalent and lethal form of brain tumor in adults.^[1]^ According to the 2016 revised World Health Organization (WHO) classification of central nervous system tumors, glioblastoma (GBM) (WHO IV) accounts for approximately 50% of glioma cases.^[2]^ The majority of gliomas demonstrate resistance to all current standard treatment modalities, including surgical resection, radiotherapy, and systemic chemotherapy. Despite the administration of all conventional therapies, the majority of glioma patients ultimately experience a recurrence of their condition.^[3]^

Secretory carrier membrane proteins (SCAMPs) constitute a group of membrane transporter proteins that are found in a variety of organisms, including plants, insects, and mammals. The mammalian genome contains 5 distinct SCAMP genes, designated SCAMP1 through SCAMP5. SCAMPs are involved in the vesicular recycling fusion of vesicles and cell membranes and regulate cytokinesis and endocytosis, activate synaptic function, and transmit neural signals. SCAMP protein is highly expressed in cells with secretory functions, such as leukocytes and neuronal cells.^[4]^ Among these proteins, SCAMP5 is highly expressed in the brain and directly or indirectly impacts the function of the central nervous system. SCAMP5 regulates membrane transport, controls cytokinesis, and is associated with secretory carriers and membrane function. In addition, SCAMP5 plays an important role in the normal maintenance of physiological functions of nerve cells.^[5]^ Also, SCAMP has been linked to the development and progression of several human cancers. It has been shown that SCAMP5 expression is an independent prognostic factor in pancreatic cancer and that it is downregulated in pancreatic cancer.^[6]^ However, in acute myeloid leukemia, SCAMP5 expression is elevated.^[7]^

The role of SCAMP5 in glioma remains unclear. One study explored the clinical significance of individual genes in gliomas and developed a prognostic model through an online database.^[8]^ This paper presents our comprehensive analysis of the relationship between SCAMP5 and glioma, assessing SCAMP5 expression in glioma through Chinese Glioma Genome Atlas (CGGA), gene expression profile interactive analysis (GEPIA), and database for annotation, visualization, and integrated discovery (DAVID) data. We analyzed the expression of SCAMP5 in different gliomas using the CAAG and developed a prognostic prediction model to predict patients’ survival at 1, 2, 3, 5, and 10 years.

The clinical and follow-up information for all patients included in this study is accessible on the CGGA website (http://www.cgga.org.cn/).

## 2. Methods

### 2.1. SCAMP5 analysis in the GEPIA database

In order to examine the correlation between SCAMP5 and GMB, we utilized the GEPIA database (http://gepia.cancer-pku.cn/) which integrates 8587 normal samples and 9736 tumor samples from the TCGA and GTEx projects. This database employs ribosomal RNA sequencing data developed by Peking University. Tumor and normal tissues were utilized to conduct differential expression analysis of SCAMP5.

### 2.2. Patients and cohort inclusion

The CGGA website was used to obtain 1018 patients’ follow-up and gene expression information. There were 325 patients in the training group^[9–12]^ and 693 patients in the validation group.^[9,12–14]^ According to the CGGA website, mRNA sequencing was conducted for both the training and validation datasets. Various clinical information was available for all patients, including WHO grade, gender, overall survival (OS), and review censoring, 23,961 gene expression levels were identified.

### 2.3. Cox survival analysis of the relationship between SCAMP5 gene expression and OS

The relationship between SCAMP5 gene expression and other characteristics, including age, gender, isocitrate dehydrogenase (IDH) mutation status, and OS in glioma patients, was examined. The endpoints of the Cox survival analysis were determined through a comprehensive review of the relevant literature. In light of the favorable factors identified in the univariate analysis, a multifactorial analysis was conducted.

### 2.4. Functional enrichment analysis

The most relevant genes for the SCAPM5 gene were identified through Pearson correlation analysis. Subsequently, these genes were ordered in descending order, with the top 500 genes selected for upload to the DAVID (https://david.ncifcrf.gov/). In this analysis, the official gene symbols and Homo sapiens were selected as parameters. Subsequently, Gene Ontology (GO) analyses, including GOTERM BP DIRECT (BP), GOTERM CC DIRECT (CC), and GOTERM MF DIRECT (MF), were downloaded, as were Kyoto Encyclopedia of Genes and Genomes (KEGG) pathway^[15–17]^ analysis enrichment results. In this study, the initial 6 results, ordered in ascending order of *P*-value, are presented.

### 2.5. Prognostic difference analysis

The patients in both the training and validation groups were divided into 2 groups based on their SCAMP5 gene expression levels. The grouping criterion was the mean expression value of the SCAMP5 gene. Individuals exhibiting values exceeding the mean were categorized as belonging to the high-expression group, whereas those displaying values below the mean were assigned to the low-expression group. Kaplan–Meier (K–M) curves were plotted in order to facilitate the visualization of the prognostic differences. The prognostic value was subjected to a log-rank test to ascertain its statistical significance. A *P*-value of <.05 was deemed to be statistically significant. The K–M curves were plotted in the statistical software R.

### 2.6. Construction of the predictive OS rate model

The prediction model was constructed using 6 factors, specifically polygenic risk score (PRS) type, age, grade, IDH mutation status, 1p/19q codeletion status, and SCAMP5 gene expression level. The total and points sum of each factor could predict the patients’ survival rates for 1, 2, 3, 5, and 10 years. The accuracy of the model was demonstrated through the use of calibrated curves and C-index values.

### 2.7. Statistical analysis and plots

Cox survival analyses were conducted using the IBM SPSS Statistics software. (version 26, Chicago, IL). GSVA analyses were conducted within the R environment, utilizing the GSVA package. GO and KEGG pathway enrichment analyses were conducted using the DAVID (https://david.ncifcrf.gov/, version 6.8). The comprehensive gene lists from the GO and the KEGG were obtained from the website (http://download.baderlab.org/EM_Genesets/current_release/Human/symbol). For all statistical methods, a *P*-value of <.05 was considered statistically significant. All heat maps, histograms, scatter plots, and calibration plots were generated using the R statistical computing environment (https://www.r-project.org/ version 4.2.2).

## 3. Results

### 3.1. SCAMP5 analysis in the GEPIA database

**Figure 1.**
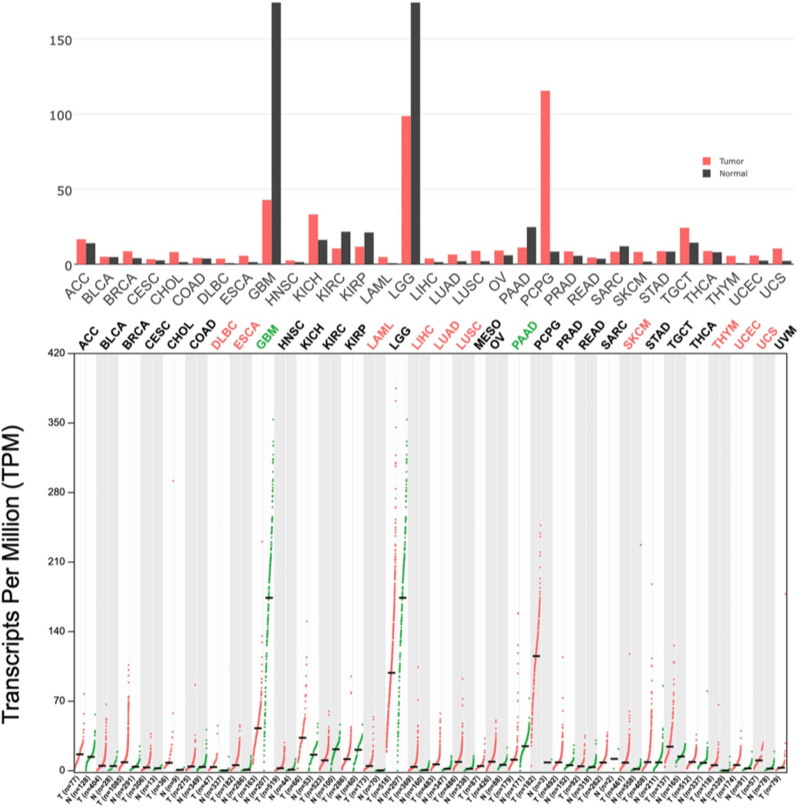
The gene expression profile across all tumor samples and paired normal tissues. The height of bar represents the median expression of certain tumor type or normal tissue. Each dot represents an expression of samples. SCAMP5 was found to be highly expressed in normal brain tissues and had lower expression in GBM.

The expression levels of SCAMP5 in GBM and normal human brain tissues were then compared using GTEx data. SCAMP5 gene is differentially expressed in different malignant tumors. SCAMP5 was highly expressed in normal brain tissues and lowly expressed in GBM (Fig. 1).

### 3.2. Expression of the SCAMP5 gene is correlated with the prognosis of glioma patients

**Figure 2.**
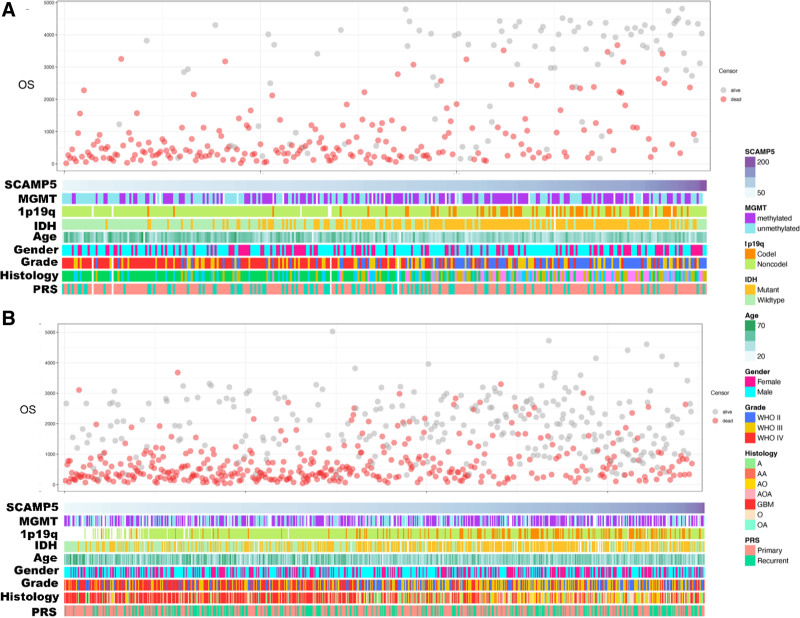
The relationship between the expression value of the SCAMP5 gene and clinical characteristics. (A) Training group. (B) Validation group. All data are arranged in ascending order of the expression value of the SCAMP5 gene.

The study involved a total of 1018 patients, and their data is presented in Figure 2. The patients are listed in ascending order of their SCAMP5 gene expression levels.

There were no significant statistically significant differences in SCAMP5 gene expression with MGMT status or PRS type in the training group. Significant statistical differences were observed in the SCAMP5 gene expression between WHO grades, IDH mutation status, and 1p/19q co-deletion groups.

**Figure 3.**
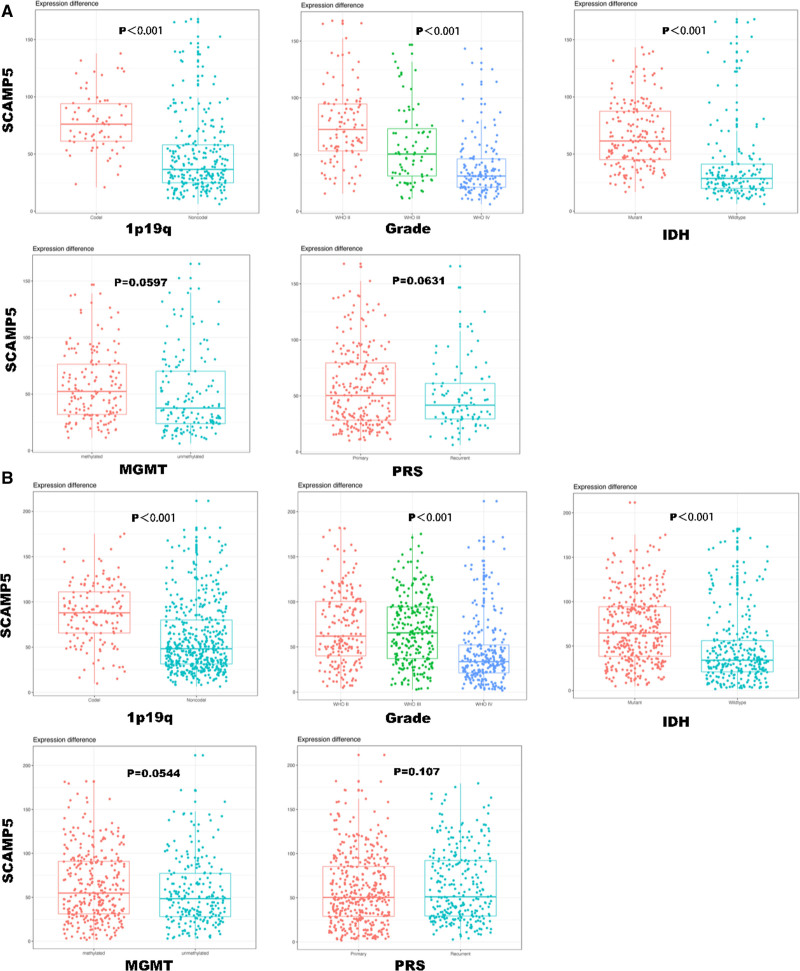
The expression value of the SCAMP5 gene with respect to various factors. (A) Training group. (B) Validation group. All differences were tested by Student *t* test, and *P* < .05 was considered statistically significant (*P < .05).

The validation group had similar results (Fig. 3).

Subsequently, we conducted an analysis to ascertain whether specific factors were significantly associated with patient survival. In the training group, significant correlations were identified between OS in glioma patients and the following factors: SCAMP5 gene expression, PRS type, age, IDH mutation status, 1p/19q co-deletion status, radiotherapy, temozolomide (TMZ) treatment, and WHO grade. No correlation was observed between gender and OS in glioma patients. Accordingly, the expression of the SCAMP5 gene, PRS type, age, IDH mutation status, 1p/19q co-deletion status, and WHO grade were incorporated into the Cox proportional risk model (Cox model). The results of our study indicate that the expression level of the SCAMP5 gene is an independent predictor of OS in patients with glioma. In the validation cohort, the expression level of the SCAMP5 gene was also identified as an independent predictor of OS in patients with glioma (Table 1).

**Table 1:** Univariate and multivariate survival analysis results of various factors in the training group and validation group.

	Univariate analysis		Multivariate analysis	
	Exp (B) (95% CI)	*P*	Exp (B) (95% CI)	*P*
*Univariate and multivariate prognostic analysis in the training group overall survival (OS*)				
PRS	2.874	0	2.756	0
Grade (1)	0.112	0	0.186	0
Grade (2)	0.393	0	0.541	.001
Gender	0.941	.66	–	–
Age	1.033	0	1.016	.022
Radio	0.632	.005	1.059	.766
Chemo	1.445	.014	0.601	.003
IDH	0.355	0	0.923	.668
1p19q	0.17	0	0.304	0
SCAMP5	0.978	0	0.990	0
*Univariate and multivariate prognostic analysis in the validation group overall survival (OS*)				
PRS	2.182	0	2.158	0
Grade (1)	0.144	0	0.169	0
Grade (2)	0.365	0	0.669	.005
Gender	1.061	.564	–	–
Age	1.026	0	1.013	.003
Radio	1.241	.109	–	–
Chemo	1.242	.081	–	–
IDH	0.323	0	0.624	.001
1p19q	0.268	0	0.440	0
SCAMP5	0.989	0	0.995	.003

### 3.3. GSVA determined the function of the SCAMP5 gene

**Figure 4.**
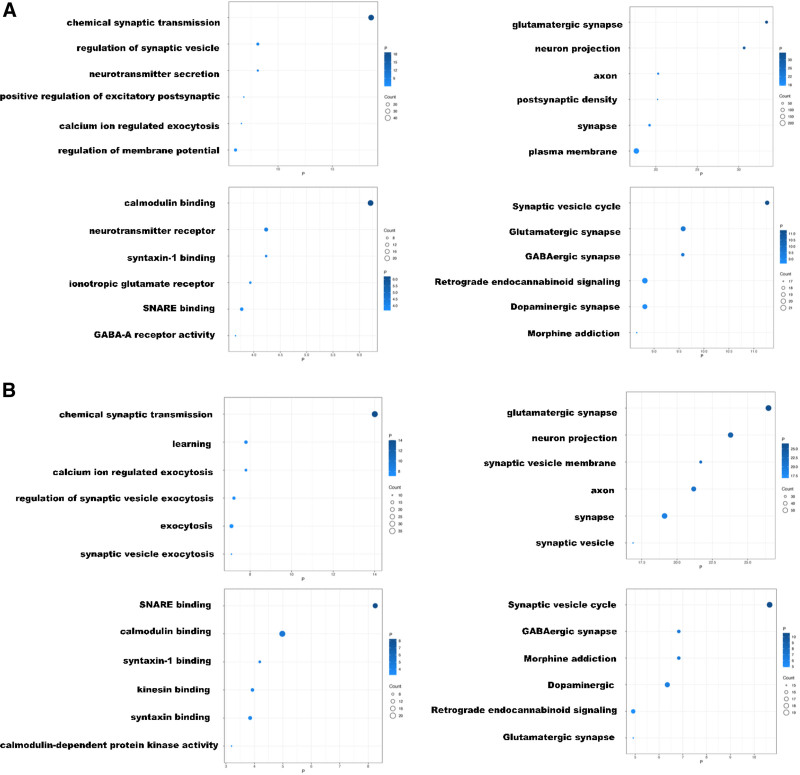
The results of biological functional enrichment analysis of the SCAMP5 gene on the DAVID website. (A) Training group. (B) Validation group.

In order to ascertain the function of the SCAMP5 gene in glioma patients, the top 500 associated genes were selected from the Pearson correlation analysis (*R* > 0.5, descending order *P* < .05) and subjected to DAVID analysis. The results of the GO and KEGG analyses described above were maintained. The results of the 6 most significant GO and KEGG analyses are presented in ascending order of *P*-value (Fig. 4).

**Figure 5.**
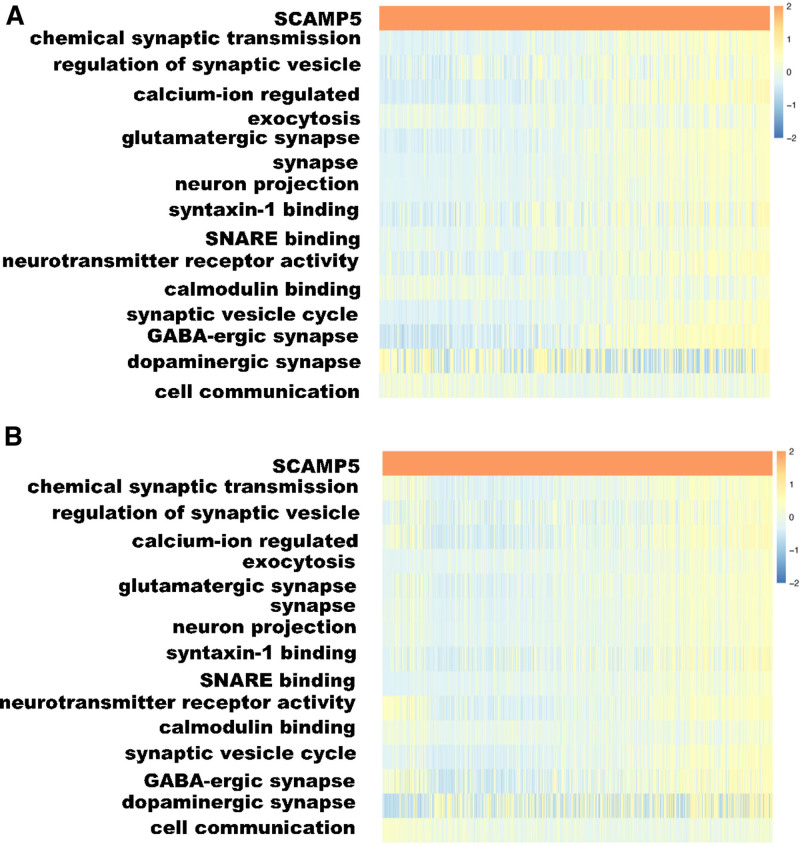
The GSVA results of SCAMP5 biological functional enrichment analysis. (A) Training group. (B) Validation group. All items are arranged in descending order of the *R*-value. The *R* values were calculated using the Pearson correlation analysis method in the R environment.

With regard to the biological processes in which the SCAMP5 gene is involved, these were found to include chemical synaptic transmission, regulation of synaptic vesicle exocytosis, and neurotransmitter secretion. With regard to cellular components, the SCAMP5 gene is predominantly localized to glutamatergic synapses and axons. With regard to molecular functions, the SCAMP5 gene is primarily engaged in calmodulin binding, neurotransmitter receptor activity, and syntaxin-1 binding. Moreover, the SCAMP5 gene is primarily implicated in the synaptic vesicle cycle, glutamatergic synapse formation, and GABAergic synapse development. The validation group yielded comparable results, thereby corroborating our findings (Fig. 5).

### 3.4. SCAMP5 may predict OS in glioma patients

**Figure 6.**
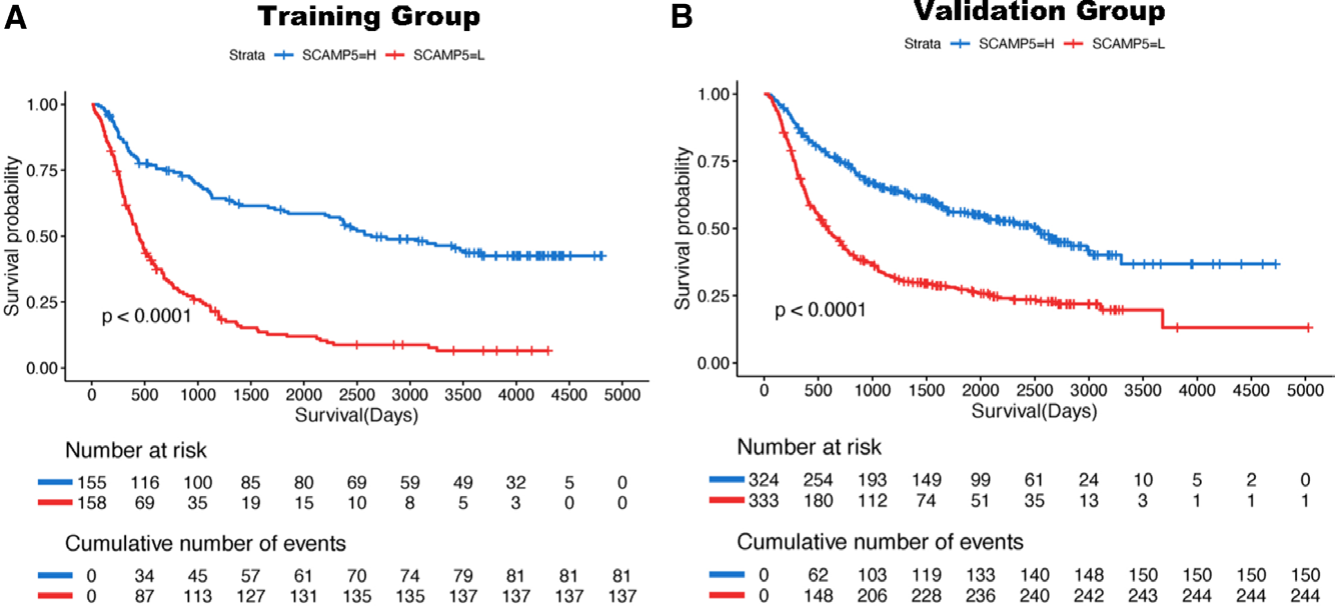
Kaplan–Meier curves (K–M curves) of the expression value of the SCAMP5 gene in each group. (A) Training group. (B) Validation group. All NA values were deleted.

In order to ascertain whether the SCAMP5 gene can be used to predict the prognosis of patients with glioma, we performed K–M and Cox proportional hazard model analyses of the training and validation groups. Patients with elevated SCAMP5 gene expression exhibited prolonged OS. The validation group yielded analogous results (Fig. 6).

### 3.5. Prediction model accurately predicts the OS of glioma patients

**Figure 7.**
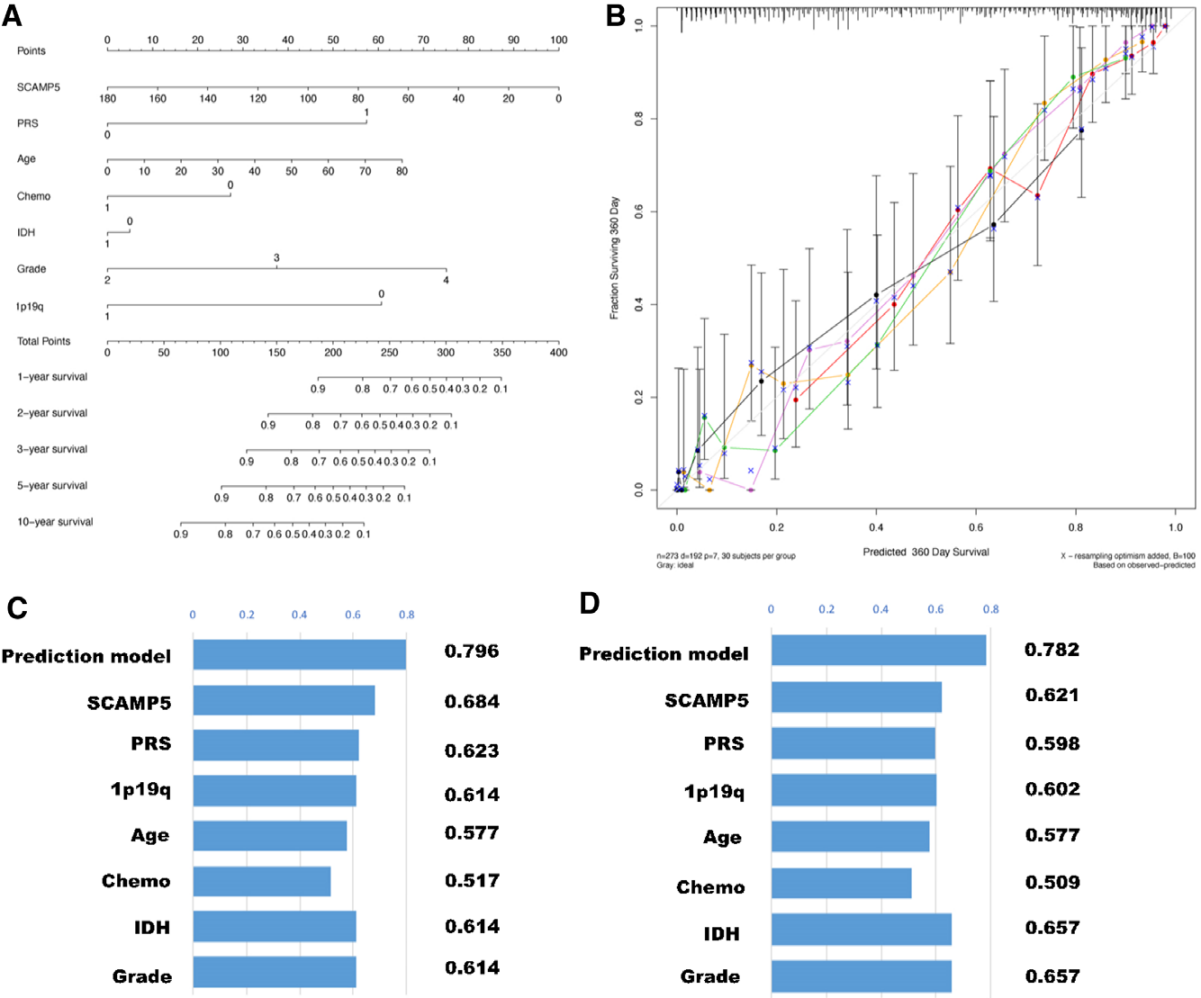
(A) The prediction model of the training group. (B) The calibration curve. (C) The C-index of the training group prediction model. (D) The C-index of the validation group prediction model.

To further assess the predictive capacity of SCAMP5 gene expression, we constructed a prediction model to estimate OS in glioma patients. The prediction model was constructed on the basis of the independent predictive factors employed in the multivariate Cox survival analyses. The aforementioned factors include the PRS type, WHO grade, IDH mutation status, 1p/19q co-deletion status, and SCAMP5 gene expression (Fig. 7).

The prediction model is capable of forecasting the OS of glioma patients at 1-, 2-, 3-, 5-, and 10-year intervals. The model enables the calculation of individual factor scores for glioma patients, which can then be combined to obtain a total score. The OS rates of patients can be predicted at 1-, 2-, 3-, 5-, and 10-year intervals using this total score. The calibration curve and C-index demonstrate that the prediction model exhibits robust predictive capability.

The C-index of the training group’s prediction model is 0.796, which is higher than that of the other single factors. The C-index for the validation group’s prediction model was 0.782, which also surpassed that of the other single factors. Furthermore, a prediction model was constructed for the validation group. Figure 1S, Supplemental Digital Content, http://links.lww.com/MD/O251 illustrates the prediction model and calibration curve for the validation group.

## 4. Discussion

Among tumors of the central nervous system, glioma is the most common type of tumor in the central nervous system. In the WHO’s classification of malignancy, a higher grade means greater difficulty in treatment and is more likely to recur. A higher grade means that it is more difficult to treat, more likely to recur, and a higher death rate.^[18]^ Therefore, an accurate assessment of the prognosis is essential to select the most appropriate treatment at an early stage of the disease to improve the patient’s prognosis. It is crucial to select the most appropriate treatment early to improve the patient’s prognosis. Studies have shown that biomarker-based cancer therapy can be effective in improving the prognosis of certain malignancies.^[19]^

SCAMP5, an important member of the SCAMP family, has received considerable attention in recent years. It is mainly distributed in the Golgi, TGN, and Golgi-derived vesicles. The structure of SCAMP5 consists of an N-terminal tail, 4 TMDs, and a C-terminal tail. The TMDs contain a 2/3 loop domain.^[20]^ The SCAMP5 gene plays an important role in a variety of brain disorders such as epilepsy, Huntington disease, and Parkinson disease.^[5]^

However, the relationship between the SCAMP5 gene and glioma patients remains to be further explored.

The GEPIA database shows significant variability in SCAMP5 expression in a few malignant tumors. In GMB multiforme and low-grade gliomas of the brain, SCAMP5 is highly expressed in normal subjects and lowly expressed in tumor patients. In pheochromocytoma and paraganglioma, SCAMP5 is highly expressed in tumor patients and lowly expressed in the normal population. This suggests that the SCAMP5 gene may be involved in and regulate the expression of neurological tumors.

By leveraging the convenience and precision offered by the CGGA database in collecting and organizing extensive data sets, our objective was to elucidate the correlation between SCAMP5 gene expression and glioma patients. A total of 325 patients were included in the training group, while 693 patients were included in the validation group. The objective was to ascertain whether specific factors, including age, SCAMP5 gene expression, radiotherapy, and TMZ chemotherapy, were associated with the survival prognosis of patients with glioma. The univariate analysis of the training group revealed that the expression of the scamp5 gene, the type of PRS, age, IDH mutation status, 1p19q co-deletion status and WHO grade were associated with OS in glioma patients. Conversely, gender was not found to be associated with OS. However, the results differed in the validation group. No association was found between radiotherapy and TMZ chemotherapy and patient survival prognosis. It is thought that this discrepancy may be attributable to the differing characteristics of the 2 sample groups. A multifactorial survival analysis incorporating numerous factors associated with patient prognosis revealed that SCAMP5 gene expression was also associated with patient survival prognosis. The results of the validation group also corroborated this finding. In light of these findings, we may conclude that SCAMP5 gene expression represents an independent factor capable of predicting OS.

Following confirmation of the strong association between SCAMP5 gene expression and patient survival prognosis, our objective was to gain insight into the role of the SCAMP5 gene and its expression products in glioma tumor development. The relationship between SCAMP5 gene expression and the expression of other genes was initially examined using Pearson correlation analysis. The results were then ordered in descending order of *R*-value, with the first 500 genes subjected to DAVID analysis. The analyses demonstrated that SCAMP5 genes are predominantly distributed in the Golgi apparatus and Golgi-derived vesicles, regulating the cytosolic action of synaptic vesicles. Additionally, they are associated with secretion carriers and membrane functions. However, further investigation is required to elucidate their precise functions and mechanisms.

Secondly, we observed that elevated SCAMP5 gene expression was associated with a more favorable clinical prognosis for patients. Increased SCAMP5 gene expression was accompanied by an increase in OS for patients.

The expression of the SCAMP5 gene is correlated with the OS of patients, and the objective was to construct a model for predicting survival in patients with gliomas. The model was constructed using 6 factors (SCAMP5 gene expression, Age, PRS type, IDH mutation status, WHO grade, and 1p/19q co-deletion status), which were identified as independent predictors. The presence of IDH mutations was found to be associated with a favorable prognosis in patients with glioma, particularly in those with high-grade glioma.^[21,22]^ In high-grade gliomas, co-deletion of 1p/19q was associated with improved treatment response and survival.

Our forecasting model’s C-index was 0.796, a favorable result, indicating that our prediction model is capable of accurately estimating the OS of patients. In order to validate the model, 6 additional factors were considered: SCAMP5 gene expression, age, type of PRS, IDH mutation status, WHO grade, and 1p/19q co-deletion status.

The predictive model in the validation group had a C index of 0.782, slightly higher than that of the training group, indicating that the constructed model can accurately predict OS. The results from the 2 groups are very similar and provide strong evidence that the predictive model developed can predict the outcome of glioma patients.^[23,24]^

The present study is limited by the small sample size, which has resulted in inconsistent proportions of various predictors and a lack of uniformity in the criteria employed for building predictive models. More extensive clinical studies are necessary to develop precise predictive models. Another limitation is the requirement for more tests to verify our results. Furthermore, radiotherapy and TMZ chemotherapy were the standard treatment in various studies. Further research is necessary to establish why radiotherapy was not crucial in our study.

In summary, SCAMP5 is significant in preserving the regular maintenance of the neurological functions of nerve cells. Impaired expression or function of SCAMP5 partly contributes to the abnormal synaptic function and hindered clearance of neurotoxic proteins, leading to potential neurological disorders. A significant association exists between the expression of the SCAMP5 gene and the survival of patients, where high expression of this gene is indicative of a positive clinical prognosis. Ultimately, we formulated a prediction model that would effectively forecast the survival of patients.

## Author contributions

**Conceptualization:** Ling Zeng, Xianbi Tang, Yushi Zhong.

**Supervision:** Li Liu.

**Writing – original draft:** Bin Wu.

**Writing – review & editing:** Yushi Zhong.
